# A Co-production Values and Principles Compass to Guide Along the Underused Pathway: Comment on "Research Coproduction: An Underused Pathway to Impact"

**DOI:** 10.34172/ijhpm.8835

**Published:** 2024-12-21

**Authors:** Daniel Masterson, Lynn Laidlaw

**Affiliations:** ^1^The School of Health Sciences, University of Skövde, Skövde, Sweden.; ^2^Jönköping Academy for Improvement of Health and Welfare, School of Health and Welfare, Jönköping University, Jönköping, Sweden.; ^3^Centre for Health and Development, University of Staffordshire, Stoke-on-Trent, UK.; ^4^Patient and Public Partner, Whitley Bay, UK.

**Keywords:** Co-production, Values, Knowledge Co-production, Research Co-production, Integrated Knowledge Translation, Implementation

## Abstract

Co-production in research is not only encouraged but rapidly becoming a required consideration in health research funding. The challenge in defining co-production continues and the misapplication of co-production has led to growing calls for an emphasis on operationalising the values and principles of co-production in research. This commentary considers Rycroft-Malone and colleagues’ key messages about co-production being more than a set of activities, and reflects on the challenges within the academic sector when applying co-production. The Co-producing Meaningful Principles and Sharing Standards (Co-MPASS) tool offers a way to consider co-production values in the early stages of collaboration. Rather than a stand-alone tool for co-production, it is intended to be used with established methods and published toolboxes to emphasise co-production principles through reflection, conversation, documentation, and learning.

## Background

 There have been recent calls for a shift towards an emphasis on the application of values and principles of co-production in research and practice.^1-3^ Norström et al^4^ suggest four principles, in that knowledge co-production should be *context-based, pluralistic, goal-orientated, *and* interactive*. In their rapid review on co-production values within research, O’Mara-Eves et al^3^ call for consideration of four core values: *human and personal, transparent, inclusive, *and* challenging*. As is becoming evident, the co-production values in a research context are increasingly documented and defined. Rycroft-Malone et al^5^ succinctly outline the principles of genuine research co-production and call on knowledge user and researchers to embrace this way of thinking. This commentary supports Rycroft-Malone and colleagues’^5^ key messages and reflects on the argument that research landscapes, current structure, governance and policy frameworks need to evolve. Rycroft-Malone et al^5^ note that research co-production is not aligned with any one method and suggest that systematizing research co-production will increase uptake and call for the continued development of the research co-production toolbox. While encouraging their call for shared learning and implementation of co-production values, our dialogue when writing this editorial centred around the research co-production toolbox. During this dialogue, Lynn Laidlaw observed that the academic focus on methods, processes and need to produce an outcome will inevitably lead to an increase of toolboxes for co-production, yet these perpetuate the current academic structure. The Patient Experience Library^6^ found over 500 toolkits for Patient and Public Involvement and report that between 2016 and 2020, toolkits were being published at an average rate of one per week. Yet a synthesis from Greenhalgh et al^7^ indicates such toolkits were rarely used beyond the groups which develop them. Lynn agrees with Rycroft-Malone et al^5^ that genuine co production is a relational way of working, guided by values and principles, founded on relationships, conversations and collaboration. In In Lynn’s experience, academics want to know how to “do” co-production, whereas co-production is as much about the “being,” which can be messy, emotional and relies on soft skills which cannot be captured by tools. Further, in line with points raised by and Rycroft-Malone et al,^5^ co-production is more than a set of activities, it is fundamental and epistemological shift in knowledge production and the challenges within academic sector need to be addressed to reflect this.^8^ Daniel Masterson agrees with the points raised by Lynn, particularly with experiencing the academic drive to produce outputs. However, Daniel supports the call for development of the co-production toolbox, noting the distinction from a “toolkit,” as a range of “tools” which can be applied in different contexts to help to guide genuine co-production. The challenge is not only how we encourage this way of thinking, but how we prevent toolboxes from becoming “tickboxes,” without first evolving the current academic culture in which co-production has to operate. Our suggestion is for a transferable, guiding tool to establish the *intention* for genuine co-production in order to help navigate this underused pathway to impact.

## Ways of Working

 Lynn Laidlaw identifies as a researcher with an interest in the values and principles and processes of co-production. Daniel Masterson has extensive experience as a public contributor on research projects, including being a co-investigator and peer researcher conducting qualitative research. They were part of the team who co-produced a rapid critical review into the value of co-production.^3^ The author’s collaboration began in 2019, when they met to discuss the term “empowerment” and the connotations inferred (who gives power to whom). This coincided with analysis of over 1000 definitions of co-production and led to dialogue between the authors on how few had any reference to co-production values. Through* conversations* and *reflection of power imbalances* and *overcoming barriers* created by current academic structures, the authors agreed to collaborate on an “as and when possible” basis. The authors met informally in their free-time, once every two-three months and had conversation on the topic of co-production. The foundation for our way of working was that we were *equal but different*, and that we were both *accountable* to the collaborative process. Each author *shared and valued knowledge from different perspectives* and it was through dialogue and sharing these *assets*, with careful consideration of *power dynamics*, which *built a collaborative and meaningful relationship*. During each video conference, we reviewed a selection of extracted definitions using a digital whiteboard. The definitions were sourced from a scoping review which identified the sixty most commonly cited definitions of co-production and co-design in the context of health and social care.^1^ Lynn would comment on the definition wording, highlight themes related to values and principles, and consider the application of these definitions in practice with a critical and reflective perspective. For example, one observation was that popular co-production definitions had rarely been co-produced. Daniel would document notes while both authors thematically analysed the definitions, with drafts themes added during the meeting and in-between meetings. At the start and end of each meeting, themes and notes were reviewed for interpretation and clarification. The *value created* through this *reciprocal partnership* was a deeper, *mutual understanding* of our challenges and experiences in co-producing and insight into practical solutions through operationalisation of co-production values. These conversations and continued ways of working led to the development of the co-production values compass which is the basis of this manuscript. Upon reading Rycroft-Malone et al,^5^ we wanted to support their message and communicate this tool to apply the values and principles in knowledge coproduction.

## Developing a Tool to Guide Us

 From analysis of the sixty most common definitions, we identified 164 separate descriptions of values. This process was repeated for articles located by a more recent rapid review^3^ which identified an additional 39 descriptions of values. Descriptions of values and preliminary themes were then added into a Microsoft Excel database and were screened for duplications (eg, same theme, same citation) and have informed the description of each value in Table. These were then reviewed and dialogue took place between the authors to review themes, leading to the identification of forty values for co-production (Table). These values were sourced from 57 different citations published between 1981 and 2022 (See Supplementary file 1). The wording and description for each value was first informed by the original extracted definitions, then written as verbs to emphasise “being” and “doing” and to be more accessible.

**Table:** Values for Co-production

	**Value**	**Description**	**Source**^a^
1	Address emotions	Exploring the emotional journey, feelings and experiences and conveying these in an impactful way.	4, 8, 34
2	Address equity	Equality, equity & fairness: Necessitating a shift in power so that no one group, person or experience is more or less important.	8, 9, 10, 15, 44, 52, 54
3	Address justice	Support social justice with the intention of creating a fairer and more equitable society.	14, 17
4	Address mutuality	Mutual sharing of feelings, action, and responsibility.	7, 36, 52, 54
5	Address power dynamics	Understanding power differentials across individual, interpersonal and structural levels and reallocate power.	15, 19, 22, 23, 24, 39, 48, 52, 54, 58
6	Be active in partnership	Establish active and effective interpersonal skills and partnerships.	12, 13, 20, 21, 36, 44, 46, 48, 49, 58
7	Be flexible	Requiring a substantial degree of nimbleness and adaptability from all involved.	16, 28, 31
8	Be genuine	Meaningful, authentic and transparent interactions that go beyond simply ‘representation’ or one-way knowledge gathering.	22, 28
9	Be human & personal	Human - value people as people, do everything wholeheartedly, and work to make a genuine difference.	18
10	Be inclusive	Meetings, materials and infrastructure are accessible so everyone can be included and engage fully.	18, 21, 24, 35, 52, 58
11	Being prepared to *act*	Coming together to find shared solutions. Turning ideas into action.	8, 18, 38, 43, 52
12	Blur boundaries	Avoid “them and us,” remove distinctions, blur boundaries and break down the barriers.	21, 26, 38, 44, 48, 52, 54
13	Build capacity	Building on people’s existing capabilities	23, 52, 54
14	Build on strengths	Emphasise capacity, strengths and assets of people.	6, 7, 14, 35, 36, 52, 54
15	Build relationships	Building relationships, supporting each other and meeting of minds.	3, 6, 7, 8, 28, 32, 41, 48, 52, 53
16	Challenge	Challenging power exploitation, question status quo and existing paradigm.	17, 18, 28, 31, 40
17	Commit the time	Creating an environment where frequent interactions can occur throughout all levels and stages of an ongoing process.	15, 28, 31, 38, 44
18	Communicate	Clear and effective, two-way communication. Listen to all voices.	3, 15, 37
19	Create change	Helping to become effective agents of change.	9, 10, 18, 52
20	Create choice	Open doors, voluntary efforts and exit opportunities.	1, 11, 12, 42, 45, 56
21	Create value	Emphasis on the outcomes in which all involved hold value.	2, 12, 13, 20, 42, 47, 52, 53, 55
22	Decide together	Making decisions openly and collectively.	18, 26, 38, 44, 48, 51, 57
23	Diversify	Value diversity in range of voices, perspectives, knowledge, culture and experiences.	30, 52
24	Doing *with* not *for*	Working WITH rather than working FOR.	22, 33
25	Enable	Enabling and facilitating rather than delivering or directing.	39, 54
26	Engage in dialogue	Learning together through continuous dialogue and reflection.	3, 21
27	Learning	Mutual learning for all people involved.	23
28	Reasoning	Share and understand the different reasons why people have engaged.	1, 19, 31, 39, 47
29	Reciprocate	Ensuring that people receive something back for putting something in.	14, 23, 36, 52, 53, 54
30	Reflect	Reframing knowledge and maximising reflexivity to transform practice.	25, 26, 28, 50
31	Respect	Showing mutual respect for each other’s roles and contributions.	52
32	Share goals	Establishing a common purpose and identifying a set of clear, shared goals and actions.	3, 15
33	Share knowledge	Sharing personal, experiential knowledge as well as diverse, professional knowledge.	4, 5, 8, 15, 17, 34, 38, 48, 49, 51, 54
34	Share leadership	Equal access to information, shared leadership and agree who defines outcomes and how.	4
35	Share ownership	Generate collective ownership, understanding and support by all.	19
36	Share understanding	Understand different ways of interacting and arriving at a shared understanding.	23
37	Take responsibility	Agree on how responsibility is organised and being accountable.	3, 15, 18, 41, 53
38	Trust	Building trusting relationships and an ability to trust.	7, 14, 37, 41
39	Value all contributions	Value, recognise and respect all people's opinion and contributions.	15, 28, 29, 48, 52
40	Work together	Transdisciplinary rather than mono- or multidisciplinary.	25, 26

^a^See Supplementary file 1.

 With consideration of the very values we were analysing, it was clear that it was not the authors role nor right to choose which values should be communicated as the most relevant for co-production. Further, the relevance of each value will change depending on the context in which co-production takes place. We present these values alphabetically and encourage that when embarking on a co-production process, that these are chosen through dialogue and a shared decision. The number of articles reported in Table should also not be considered an indication of importance. These values can be adapted or reduced based on the groups needs and discussions. Further, we encourage the addition of values raised during the collaboration, which may be missing or identified in future research.

## When Values Become Principles

 Schwartz^9^ defines values as “beliefs and desirable goals” that motivate action which “transcend specific actions and situations” (p. 4) and can serve as standards, criteria or guidelines. The purpose of the Co-MPASS (Co-producing Meaningful Principles and Sharing Standards) presented in Figure is to guide planning and implementing co-production values throughout the co-production process. As noted by Knowles et al,^10^ co-production is more than a method, it is a way of working requiring space to talk and space to change. The compass emphasises the need for *dialogue, documentation, reflection*^11^ and *learning*^12^ in order to create this space. As needs will differ from group to group and in different contexts, our suggestion for implementation below can be adapted to meet the needs of the group.

**Figure:**
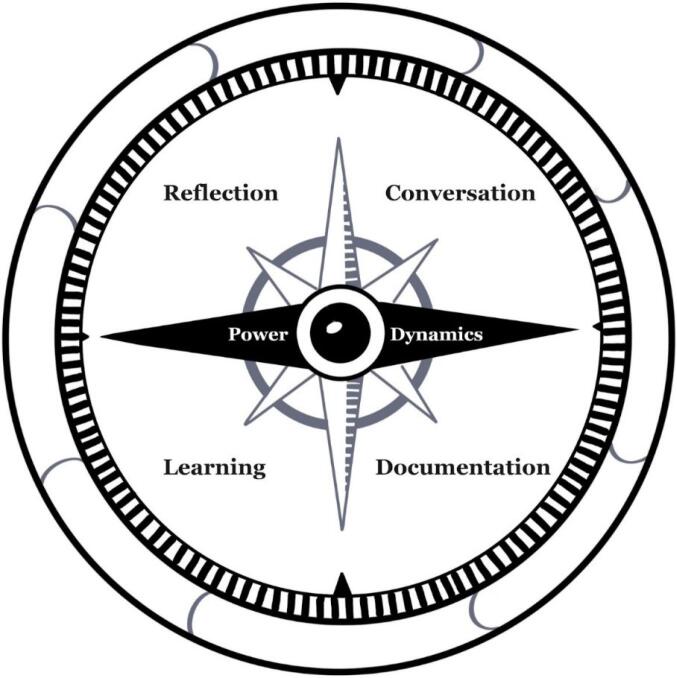
The Co-producing Meaningful Principles and Sharing Standards Tool.

*Individual reflection:* Each person considers the values described in Table and are asked to reflect on which they feel are most important to them. Individuals can choose to combine, adjust or add values to make them relevant to their own situation and experience. Each person selects four to six values to discuss.

*Conversations*: Each person takes turns to explain why a particular value is important to them. If the next person has chosen the same value, they highlight this and then describe another value. The process continues until all of the chosen values have been discussed by the group. These are then compiled together and dialogue continues on what these values mean to each person, including those who did not choose them. This dialogue is essential and encouragement of differences of opinions in a safe environment is necessary. It should be emphasised that there are no right and wrong opinions.

*Documentation: *When the group feels that the discussion on the highlighted values has reached a natural conclusion, a decision can be made to establish which values will become the groups guiding co-production principles. How this is done should be decided by the group. The chosen values are documented onto the Co-MPASS and these become the shared co-production principles and standards for the group.

*Shared learning:* It is recommended that the group explore proposed ways of working with reflection on how these guiding co-production principles will be operationalised throughout the co-production process. We purposefully avoid recommending a specific process as the group needs to agree ways of working together, in context.

*Continued dialogue, learning, and reflection:* It is envisaged that on the co-production journey, that conversation and reflection on the guiding principles can be used to help with shared decisions, such as when coming to an important decision. This may allow for a range of possible paths (processes) to be followed, with the co-production principles and power dynamics compass needle acting as a reminder and reflective tool.

## Discussion

 This tool provides a way to begin to operationalise the values and principles of co-production by promoting dialogue on which values are most relevant to those involved in a specific context. The chosen principles can then be considered within application of a range of participatory approaches or when choosing co-production tools which have already been published. On the Co-production Collective resource website^13^ there are 43 toolkits and 23 guides available for various contexts. Notably, there are already several published tools adopting the journey metaphor of a compass. Graffigna et al^14^ provide an analytical Co-production Compass for monitoring and evaluating patient preferences of care pathways in context of mental health. Mulvale et al^15^ published a COMPASS and MAPS (Supporting **M**anagement; Building **A**ffinity; Preparing **P**articipants; Fostering **S**ensitivity) tool to assist researchers in navigating complex power relationships in the context of working with vulnerable populations. Schneider et al^16^ provide a strategic “network compass” to foster self-reflection and learning within and between interdisciplinary networks. How the Co-MPASS tool differs is that it intends to prompt reflection, selection and operationalisation of co-production values for any path chosen by those seeking authentic collaborative group work, in any context.

 It is important to note that these definitions were informed by an analysis of extracted definitions in the context of health and social care. As the extracted definitions were analysed rather than the full article, authors who may have contributed to developing the included values may have not been captured in this analysis. Further, there are likely more relevant values which have not been captured. For example, Rycroft-Malone et al^5^ discuss *scientific humility* and a *commitment to reconciliation* which were not values identified in our data set. Therefore, we do not claim the values described in Table to be an exhaustive list. Rather, it is hoped that when this tool is implemented that there is opportunity to shape, reword, combine, add, distinguish, and refine the chosen co-production values so that these are relevant to the specific context. It is hoped that this is achieved through consideration of power dynamics with conversation, documentation, reflection, and shared learning.

## Ethical issues

 Not applicable.

## Conflicts of interest

 Authors declare that they have no conflicts of interest.

## Supplementary files

Supplementary file 1. Citation Sources for Synthesised Co-production Values.
