# Supplementary material for: A Co-production Values and Principles Compass to Guide Along the Underused Pathway: Comment on "Research Coproduction: An Underused Pathway to Impact"
Source: Int J Health Policy Manag. 2024 Dec 21;13:8835. doi: 10.34172/ijhpm.8835 (PMC11806216; doi:10.34172/ijhpm.8835)
Supplement: Supplementary file 1 — Citation Sources for Synthesised Co-production Values. [file ijhpm-13-8835-s001.pdf]

Article title: A Co-production Values and Principles Compass to Guide Along the Underused Pathway; Comment on "Research Coproduction: An Underused Pathway to Impact"

Authors' information: Daniel Masterson[1,2,3]*¶, Lynn Laidlaw[4]¶

[1]The School of Health Sciences, University of Skövde, Skövde, Sweden.

[2]Jönköping Academy for Improvement of Health and Welfare, School of Health and Welfare, Jönköping University, Jönköping, Sweden.

[3]Centre for Health and Development, University of Staffordshire, Stoke-on-Trent, UK.

[4]Patient and Public Partner, Whitley Bay, UK.

***Correspondence to:** Daniel Masterson; Email: daniel.masterson@his.se

Citation: Masterson D, Laidlaw L. A co-production values and principles compass to guide along the underused pathway: Comment on "Research coproduction: an underused pathway to impact." Int J Health Policy Manag. 2024;13:8835. doi:10.34172/ijhpm.8835

Supplementary file 1. Citation Sources for Synthesised Co-production Values

Value ref.	Value label as a verb	Value label	Ref code	Full citation
1	Address emotions	Emotion	4	Bate, P., & Robert, G. (2006). Experience-based design: from redesigning the system around the patient to co-designing services with the patient. BMJ quality & safety, 15(5), 307-310.
1	Address emotions	Emotion	8	Boyd H, McKernon S, Mullin B, Old A. (2012). Improving healthcare through the use of co-design. *NZ Med J*;125(1357):76-87.
1	Address emotions	Emotion	34	The Point of Care Foundation. EBCD: Experience-based co-design toolkit. The Point of Care Foundation. Accessed July 14, 2020. https:// www.pointofcarefoundation.org.uk/resource/experience-based-codesign-ebcd-toolkit
2	Address equity	Equality and equity	8	Boyd H, McKernon S, Mullin B, Old A. (2012). Improving healthcare through the use of co-design. *NZ Med J*;125(1357):76-87.
2	Address equity	Equality and equity	9	Boyle, D., & Harris, M. (2009). The challenge of co-production. London: new economics foundation, 56(18).
2	Address equity	Equality and equity	10	Boyle D, Slay J, Stephens L. (2010). Public Services Inside Out. Putting Coproduction into Practice. NESTA.
2	Address equity	Equality and equity	15	Clark, M. (2015). Co-production in mental health care. Mental Health Review Journal, 20(4), 213-219.

2	Address equity	Equality and equity	44	Perkins, R., Repper, J., Rinaldi, M., & Brown, H. (2012). 1. Recovery colleges. Centre for Mental Health London.
2	Address equity	Equality and equity	52	SCIE (2013, 2022) Co-production: what it is and how to do it. https://www.scie.org.uk/co-production/what-how (accessed November 21st 2024).
2	Address equity	Equality and equity	52	SCIE (2013, 2022) Co-production: what it is and how to do it. https://www.scie.org.uk/co-production/what-how (accessed November 21st 2024).
2	Address equity	Equality and equity	54	Slay, J., & Stephens, L. (2013). Co-production in mental health: A literature review. London: new economics foundation, 4, 1-36.
3	Address justice	Social justice	14	Cahn, E. S. (2000). No more throw-away people: The co-production imperative. Edgar Cahn.
3	Address justice	Social justice	17	Cooper, F., & Jones, C. (2022). Co-production for or against the university: student loneliness and the commodification of impact in COVID-19. Qualitative research journal, 22(1), 81-95.
4	Address mutuality	Mutuality	7	Bovaird, T. (2007). Beyond engagement and participation: User and community coproduction of public services. Public administration review, 67(5), 846-860.
4	Address mutuality	Mutuality	36	Needham, C., & Carr, S. (2009). SCIE research briefing 31: co-production: an emerging evidence base for adult social care transformation. London: Social Care Institute for Excellence.
4	Address mutuality	Mutuality	52	SCIE (2013, 2022) Co-production: what it is and how to do it. https://www.scie.org.uk/co-production/what-how (accessed November 21st 2024).
4	Address mutuality	Mutuality	54	Slay, J., & Stephens, L. (2013). Co-production in mental health: A literature review. London: new economics foundation, 4, 1-36.
5	Address power dynamics	Power	15	Clark, M. (2015). Co-production in mental health care. Mental Health Review Journal, 20(4), 213-219.
5	Address power dynamics	Power	19	Donetto, S., Pierri, P., Tsianakas, V., & Robert, G. (2015). Experience-based co-design and healthcare improvement: realizing participatory design in the public sector. The Design Journal, 18(2), 227-248.
5	Address power dynamics	Power	22	Durose C, Beebeejaun Y, Rees J, Richardson J, Richardson L. Connected communities: towards co-production in research with communities. 2011. Accessed November 21, 2021. https://ahrc.ukri.org/documents/project-reports-and-reviews/connected-communities/towards-co-production-in-research-with-communities
5	Address power dynamics	Power	23	Egid, B. R., Roura, M., Aktar, B., Quach, J. A., Chumo, I., Dias, S., ... & Ozano, K. (2021). ‘You want to deal with power while riding on power’: global perspectives on power in participatory health research and co-production approaches. BMJ global health, 6(11), e006978.
5	Address power dynamics	Power	24	Fotaki, M. (2011). Towards developing new partnerships in public services: Users as consumers, citizens and/or co-producers in health and social care in England and Sweden. Public administration, 89(3), 933-955.

5	Address power dynamics	Power	39	Osborne, S. P., & Strokosch, K. (2013). It takes Two to Tango? Understanding the C o-production of Public Services by Integrating the Services Management and Public Administration Perspectives. British Journal of Management, 24, S31-S47.
5	Address power dynamics	Power	48	Realpe, A., & Wallace, L. (2010). What is Co-production? The Health Foundation.
5	Address power dynamics	Power	52	SCIE (2013, 2022) Co-production: what it is and how to do it. https://www.scie.org.uk/co-production/what-how (accessed November 21st 2024).
5	Address power dynamics	Power	54	Slay, J., & Stephens, L. (2013). Co-production in mental health: A literature review. London: new economics foundation, 4, 1-36.
5	Address power dynamics	Power	58	Voorberg, W. H., Bekkers, V. J., & Tummers, L. G. (2015). A systematic review of co-creation and co-production: Embarking on the social innovation journey. Public management review, 17(9), 1333-1357.
6	Be active in partnership	Partnerships	12	Brudney, J. L., & England, R. E. (1983). Toward a definition of the coproduction concept. Public administration review, 59-65.
6	Be active in partnership	Partnerships	13	Brudney, J. L. (1983). The evaluation of coproduction programs. Policy Studies Journal, 12(2), 376-385.
6	Be active in partnership	Partnerships	20	Donetto, S., Tsianakas, V., & Robert, G. (2014). Using Experience-based Co-design (EBCD) to improve the quality of healthcare: mapping where we are now and establishing future directions. London: King's College London, 5-7.
6	Be active in partnership	Partnerships	21	Dunston, R., Lee, A., Boud, D., Brodie, P., & Chiarella, M. (2009). Co-production and health system reform–from re-imagining to re-making. Australian Journal of Public Administration, 68(1), 39-52.
6	Be active in partnership	Partnerships	36	Needham, C., & Carr, S. (2009). SCIE research briefing 31: co-production: an emerging evidence base for adult social care transformation. London: Social Care Institute for Excellence.
6	Be active in partnership	Partnerships	44	Perkins, R., Repper, J., Rinaldi, M., & Brown, H. (2012). 1. Recovery colleges. Centre for Mental Health London.
6	Be active in partnership	Partnerships	46	Pestoff, V. A., Brandsen, T., & Verschuere, B. (Eds.). (2012). New public governance, the third sector and co-production (Vol. 7). London: Routledge.
6	Be active in partnership	Partnerships	48	Realpe, A., & Wallace, L. (2010). What is Co-production? The Health Foundation.
6	Be active in partnership	Partnerships	49	Robert, G., Cornwell, J., Locock, L., Purushotham, A., Sturmey, G., & Gager, M. (2015). Patients and staff as codesigners of healthcare services. Bmj, 350.
6	Be active in partnership	Partnerships	58	Voorberg, W. H., Bekkers, V. J., & Tummers, L. G. (2015). A systematic review of co-creation and co-production: Embarking on the social innovation journey. Public management review, 17(9), 1333-1357.
7	Be flexible	Flexibility	16	Cluley, V., & Radnor, Z. (2021). Rethinking co-creation: the fluid and relational process of value co-creation in public service organizations. Public Money & Management, 41(7), 563-572.

7	Be flexible	Flexibility	28	Green, R., & Baker, C. (2022). Re-empowering into voice: experiments in organic community co-production. Community Development Journal, 57(2), 277-294.
7	Be flexible	Flexibility	31	Howarth, C., Lane, M., Morse-Jones, S., Brooks, K., & Viner, D. (2022). The 'co'in co-production of climate action: challenging boundaries within and between science, policy and practice. Global Environmental Change, 72, 102445.
8	Be genuine	Authentic	22	Durose C, Beebeejaun Y, Rees J, Richardson J, Richardson L. Connected communities: towards co-production in research with communities. 2011. Accessed November 21, 2021. https://ahrc.ukri. org/documents/project-reports-and-reviews/connected-communities/towards-co-production-in-research-with-communities
8	Be genuine	Authentic	28	Green, R., & Baker, C. (2022). Re-empowering into voice: experiments in organic community co-production. Community Development Journal, 57(2), 277-294.
9	Be human & personal	Human & personal	18	Co-Production Collective (2022) Co-creating change together: Our direction for 2020 - 2022. London: Co-Production Collective, UCL
10	Be inclusive	Inclusivity	18	Co-Production Collective (2022) Co-creating change together: Our direction for 2020 - 2022. London: Co-Production Collective, UCL
10	Be inclusive	Inclusivity	21	Dunston, R., Lee, A., Boud, D., Brodie, P., & Chiarella, M. (2009). Co-production and health system reform–from re-imagining to re-making. Australian Journal of Public Administration, 68(1), 39-52.
10	Be inclusive	Inclusivity	24	Fotaki, M. (2011). Towards developing new partnerships in public services: Users as consumers, citizens and/or co-producers in health and social care in England and Sweden. Public administration, 89(3), 933-955.
10	Be inclusive	Inclusivity	35	Loeffler, E., Power, G., Bovaird, T., & Hine-Hughes, F. (2013). Co-production of health and wellbeing in Scotland. Governance international.
10	Be inclusive	Inclusivity	52	SCIE (2013, 2022) Co-production: what it is and how to do it. https://www.scie.org.uk/co-production/what-how (accessed November 21st 2024).
10	Be inclusive	Inclusivity	52	SCIE (2013, 2022) Co-production: what it is and how to do it. https://www.scie.org.uk/co-production/what-how (accessed November 21st 2024).
10	Be inclusive	Inclusivity	58	Voorberg, W. H., Bekkers, V. J., & Tummers, L. G. (2015). A systematic review of co-creation and co-production: Embarking on the social innovation journey. Public management review, 17(9), 1333-1357.
11	Being prepared to *act*	Action-orientated goals	8	Boyd H, McKernon S, Mullin B, Old A. (2012). Improving healthcare through the use of co-design. *NZ Med J*;125(1357):76-87.
11	Being prepared to *act*	Action-orientated goals	18	Co-Production Collective (2022) Co-creating change together: Our direction for 2020 - 2022. London: Co-Production Collective, UCL
11	Being prepared to *act*	Action-orientated goals	38	Norström, A. V., Cvitanovic, C., Löf, M. F., West, S., Wyborn, C., Balvanera, P., ... & Österblom, H. (2020). Principles for knowledge co-production in sustainability research. Nature sustainability, 3(3), 182-190.

11	Being prepared to *act*	Action-orientated goals	43	Percy, S. L. (1984). Citizen participation in the coproduction of urban services. Urban Affairs Quarterly, 19(4), 431-446.
11	Being prepared to *act*	Action-orientated goals	52	SCIE (2013, 2022) Co-production: what it is and how to do it. https://www.scie.org.uk/co-production/what-how (accessed November 21st 2024).
12	Blur boundaries	Blur boundaries	21	Dunston, R., Lee, A., Boud, D., Brodie, P., & Chiarella, M. (2009). Co-production and health system reform–from re-imagining to re-making. Australian Journal of Public Administration, 68(1), 39-52.
12	Blur boundaries	Blur boundaries	26	Gillard, S., Simons, L., Turner, K., Lucock, M., & Edwards, C. (2012). Patient and public involvement in the coproduction of knowledge: reflection on the analysis of qualitative data in a mental health study. Qualitative Health Research, 22(8), 1126-1137.
12	Blur boundaries	Blur boundaries	38	Nowotny, H., Scott, P. and Gibbons, M. (2001), Rethinking Science, Polity Press, Cambridge.
12	Blur boundaries	Blur boundaries	44	Perkins, R., Repper, J., Rinaldi, M., & Brown, H. (2012). 1. Recovery colleges. Centre for Mental Health London.
12	Blur boundaries	Blur boundaries	48	Realpe, A., & Wallace, L. (2010). What is Co-production? The Health Foundation.
12	Blur boundaries	Blur boundaries	52	SCIE (2013, 2022) Co-production: what it is and how to do it. https://www.scie.org.uk/co-production/what-how (accessed November 21st 2024).
12	Blur boundaries	Blur boundaries	54	Slay, J., & Stephens, L. (2013). Co-production in mental health: A literature review. London: new economics foundation, 4, 1-36.
13	Build capacity	Build capacity	23	Egid, B. R., Roura, M., Aktar, B., Quach, J. A., Chumo, I., Dias, S., ... & Ozano, K. (2021). 'You want to deal with power while riding on power': global perspectives on power in participatory health research and co-production approaches. BMJ global health, 6(11), e006978.
13	Build capacity	Build capacity	52	SCIE (2013, 2022) Co-production: what it is and how to do it. https://www.scie.org.uk/co-production/what-how (accessed November 21st 2024).
13	Build capacity	Build capacity	54	Slay, J., & Stephens, L. (2013). Co-production in mental health: A literature review. London: new economics foundation, 4, 1-36.
14	Build on strengths	Assets-based	6	Bovaird, T., & Loeffler, E. (2012). From engagement to co-production: The contribution of users and communities to outcomes and public value. Voluntas: international journal of voluntary and nonprofit organizations, 23, 1119-1138.
14	Build on strengths	Assets-based	7	Bovaird, T. (2007). Beyond engagement and participation: User and community coproduction of public services. Public administration review, 67(5), 846-860.
14	Build on strengths	Assets-based	14	Cahn, E. S. (2000). No more throw-away people: The co-production imperative. Edgar Cahn.
14	Build on strengths	Assets-based	35	Loeffler, E., Power, G., Bovaird, T., & Hine-Hughes, F. (2013). Co-production of health and wellbeing in Scotland. Governance international.
14	Build on strengths	Assets-based	36	Needham, C., & Carr, S. (2009). SCIE research briefing 31: co-production: an emerging evidence base for adult social care transformation. London: Social Care Institute for Excellence.

14	Build on strengths	Assets-based	52	SCIE (2013, 2022) Co-production: what it is and how to do it. https://www.scie.org.uk/co-production/what-how (accessed November 21st 2024).
14	Build on strengths	Assets-based	54	Slay, J., & Stephens, L. (2013). Co-production in mental health: A literature review. London: new economics foundation, 4, 1-36.
15	Build relationships	Build relationships	3	Batalden, M., Batalden, P., Margolis, P., Seid, M., Armstrong, G., Opipari-Arrigan, L., & Hartung, H. (2016). Coproduction of healthcare service. BMJ quality & safety, 25(7), 509-517.
15	Build relationships	Build relationships	6	Bovaird, T., & Loeffler, E. (2012). From engagement to co-production: The contribution of users and communities to outcomes and public value. Voluntas: international journal of voluntary and nonprofit organizations, 23, 1119-1138.
15	Build relationships	Build relationships	7	Bovaird, T. (2007). Beyond engagement and participation: User and community coproduction of public services. Public administration review, 67(5), 846-860.
15	Build relationships	Build relationships	8	Boyd H, McKernon S, Mullin B, Old A. (2012). Improving healthcare through the use of co-design. *NZ Med J*;125(1357):76-87.
15	Build relationships	Build relationships	28	Green, R., & Baker, C. (2022). Re-empowering into voice: experiments in organic community co-production. Community Development Journal, 57(2), 277-294.
15	Build relationships	Build relationships	32	Joshi, A., & Moore, M. (2004). Institutionalised co-production: unorthodox public service delivery in challenging environments. Journal of development studies, 40(4), 31-49.
15	Build relationships	Build relationships	41	Osborne, S. (2010).The New Public Governance. New York, NY: Routledge.
15	Build relationships	Build relationships	48	Realpe, A., & Wallace, L. (2010). What is Co-production? The Health Foundation.
15	Build relationships	Build relationships	52	SCIE (2013, 2022) Co-production: what it is and how to do it. https://www.scie.org.uk/co-production/what-how (accessed November 21st 2024).
15	Build relationships	Build relationships	53	Sharp, E. B. (1980). Toward a new understanding of urban services and citizen participation: The coproduction concept. Midwest Review of Public Administration, 14(2), 105-118.
16	Challenge	Challenging	17	Cooper, F., & Jones, C. (2022). Co-production for or against the university: student loneliness and the commodification of impact in COVID-19. Qualitative research journal, 22(1), 81-95.
16	Challenge	Challenging	18	Co-Production Collective (2022) Co-creating change together: Our direction for 2020 - 2022. London: Co-Production Collective, UCL
16	Challenge	Challenging	28	Green, R., & Baker, C. (2022). Re-empowering into voice: experiments in organic community co-production. Community Development Journal, 57(2), 277-294.
16	Challenge	Challenging	31	Howarth, C., Lane, M., Morse-Jones, S., Brooks, K., & Viner, D. (2022). The ‘co’in co-production of climate action: challenging boundaries within and between science, policy and practice. Global Environmental Change, 72, 102445.

16	Challenge	Challenging	40	Osborne, S. P., & Strokosch, K. (2013). It takes Two to Tango? Understanding the C o-production of Public Services by Integrating the Services Management and Public Administration Perspectives. British Journal of Management, 24, S31-S47.
17	Commit the time	Commitment	15	Clark, M. (2015). Co-production in mental health care. Mental Health Review Journal, 20(4), 213-219.
17	Commit the time	Commitment	28	Green, R., & Baker, C. (2022). Re-empowering into voice: experiments in organic community co-production. Community Development Journal, 57(2), 277-294.
17	Commit the time	Commitment	31	Howarth, C., Lane, M., Morse-Jones, S., Brooks, K., & Viner, D. (2022). The 'co'in co-production of climate action: challenging boundaries within and between science, policy and practice. Global Environmental Change, 72, 102445.
17	Commit the time	Commitment	38	Norström, A. V., Cvitanovic, C., Löf, M. F., West, S., Wyborn, C., Balvanera, P., ... & Österblom, H. (2020). Principles for knowledge co-production in sustainability research. Nature sustainability, 3(3), 182-190.
17	Commit the time	Commitment	44	Perkins, R., Repper, J., Rinaldi, M., & Brown, H. (2012). 1. Recovery colleges. Centre for Mental Health London.
18	Communicate	Communication	3	Batalden, M., Batalden, P., Margolis, P., Seid, M., Armstrong, G., Opipari-Arrigan, L., & Hartung, H. (2016). Coproduction of healthcare service. BMJ quality & safety, 25(7), 509-517.
18	Communicate	Communication	15	Clark, M. (2015). Co-production in mental health care. Mental Health Review Journal, 20(4), 213-219.
18	Communicate	Communication	37	Needham, C. (2008). Realising the potential of co-production: Negotiating improvements in public services. Social policy and society, 7(2), 221-231.
19	Create change	Create change	9	Boyle, D., & Harris, M. (2009). The challenge of co-production. London: new economics foundation, 56(18).
19	Create change	Create change	10	Boyle D, Slay J, Stephens L. (2010). Public Services Inside Out. Putting Coproduction into Practice. NESTA.
19	Create change	Create change	18	Co-Production Collective (2022) Co-creating change together: Our direction for 2020 - 2022. London: Co-Production Collective, UCL
19	Create change	Create change	52	SCIE (2013, 2022) Co-production: what it is and how to do it. https://www.scie.org.uk/co-production/what-how (accessed November 21st 2024).
20	Create choice	Choice	1	Alford, J. (2009). Engaging public sector clients: From service-delivery to co-production. Springer.
20	Create choice	Choice	11	Brandsen, T., & Pestoff, V. (2006). Co-production, the third sector and the delivery of public services: An introduction. Public management review, 8(4), 493-501.
20	Create choice	Choice	12	Brudney, J. L., & England, R. E. (1983). Toward a definition of the coproduction concept. Public administration review, 59-65.

20	Create choice	Choice	42	Parks, R. B., Baker, P. C., Kiser, L., Oakerson, R., Ostrom, E., Ostrom, V., ... & Wilson, R. (1981). Consumers as coproducers of public services: Some economic and institutional considerations. Policy studies journal, 9(7), 1001-1011.
20	Create choice	Choice	45	Pestoff, V. (2013). Co-production and third sector social services in Europe: some crucial conceptual issues. In New public governance, the third sector, and co-production (pp. 13-34). Routledge.
20	Create choice	Choice	56	Verschuere, B., Brandsen, T., & Pestoff, V. (2012). Co-production: The state of the art in research and the future agenda. Voluntas: international journal of voluntary and nonprofit organizations, 23, 1083-1101.
21	Create value	Create value	2	Alford, J. (2014). The multiple facets of co-production: Building on the work of Elinor Ostrom. Public Management Review, 16(3), 299-316.
21	Create value	Create value	12	Brudney, J. L., & England, R. E. (1983). Toward a definition of the coproduction concept. Public administration review, 59-65.
21	Create value	Create value	13	Brudney, J. L. (1983). The evaluation of coproduction programs. Policy Studies Journal, 12(2), 376-385.
21	Create value	Create value	20	Donetto, S., Tsianakas, V., & Robert, G. (2014). Using Experience-based Co-design (EBCD) to improve the quality of healthcare: mapping where we are now and establishing future directions. London: King's College London, 5-7.
21	Create value	Create value	42	Parks, R. B., Baker, P. C., Kiser, L., Oakerson, R., Ostrom, E., Ostrom, V., ... & Wilson, R. (1981). Consumers as coproducers of public services: Some economic and institutional considerations. Policy studies journal, 9(7), 1001-1011.
21	Create value	Create value	47	Ramirez, R. (1999). Value co-production: intellectual origins and implications for practice and research. Strategic management journal, 20(1), 49-65.
21	Create value	Create value	52	SCIE (2013, 2022) Co-production: what it is and how to do it. https://www.scie.org.uk/co-production/what-how (accessed November 21st 2024).
21	Create value	Create value	53	Staniszewska, S., Hickey, G., Coutts, P., Thurman, B., & Coldham, T. (2022). Co-production: a kind revolution. Research Involvement and Engagement, 8(1), 4.
21	Create value	Create value	55	Vargo, S. L., & Lusch, R. F. (2008). Service-dominant logic: continuing the evolution. Journal of the Academy of marketing Science, 36, 1-10.
22	Decide together	Shared decisions	18	Co-Production Collective (2022) Co-creating change together: Our direction for 2020 - 2022. London: Co-Production Collective, UCL
22	Decide together	Shared decisions	26	Gillard, S., Simons, L., Turner, K., Lucock, M., & Edwards, C. (2012). Patient and public involvement in the coproduction of knowledge: reflection on the analysis of qualitative data in a mental health study. Qualitative Health Research, 22(8), 1126-1137.
22	Decide together	Shared decisions	38	Nowotny, H., Scott, P. and Gibbons, M. (2001), Rethinking Science, Polity Press, Cambridge.

22	Decide together	Shared decisions	44	Perkins, R., Repper, J., Rinaldi, M., & Brown, H. (2012). 1. Recovery colleges. Centre for Mental Health London.
22	Decide together	Shared decisions	48	Realpe, A., & Wallace, L. (2010). What is Co-production? The Health Foundation.
22	Decide together	Shared decisions	51	Sanders, E. B. N., & Stappers, P. J. (2014). Probes, toolkits and prototypes: three approaches to making in codesigning. CoDesign, 10(1), 5-14.
22	Decide together	Shared decisions	57	Verschuere, B., Brandsen, T., & Pestoff, V. (2012). Co-production: The state of the art in research and the future agenda. Voluntas: international journal of voluntary and nonprofit organizations, 23, 1083-1101.
23	Diversify	Diversity	30	Habermehl, V., & Perry, B. (2021). The Risk Of Austerity Co-Production In City-Regional Governance In England. International journal of urban and regional research, 45(3), 555-571.
23	Diversify	Diversity	52	SCIE (2013, 2022) Co-production: what it is and how to do it. https://www.scie.org.uk/co-production/what-how (accessed November 21st 2024).
23	Diversify	Diversity	52	SCIE (2013, 2022) Co-production: what it is and how to do it. https://www.scie.org.uk/co-production/what-how (accessed November 21st 2024).
24	Doing WITH not FOR	With rather than for	22	Durose C, Beebeejaun Y, Rees J, Richardson J, Richardson L.<br>Connected communities: towards co-production in research with communities. 2011. Accessed November 21, 2021. https://ahrc.ukri.org/documents/project-reports-and-reviews/connected-communities/<br>towards-co-production-in-research-with-communities
24	Doing WITH not FOR	With rather than for	33	Kaulio, M. A. (1998). Customer, consumer and user involvement in product development: A framework and a review of selected methods. Total quality management, 9(1), 141-149.
25	Enable	Enabling	39	Osborne, S. P., & Strokosch, K. (2013). It takes Two to Tango? Understanding the C o-production of Public Services by Integrating the Services Management and Public Administration Perspectives. British Journal of Management, 24, S31-S47.
25	Enable	Enabling	54	Slay, J., & Stephens, L. (2013). Co-production in mental health: A literature review. London: new economics foundation, 4, 1-36.
26	Engage in dialogue	Dialogue	3	Batalden, M., Batalden, P., Margolis, P., Seid, M., Armstrong, G., Opipari-Arrigan, L., & Hartung, H. (2016). Coproduction of healthcare service. BMJ quality & safety, 25(7), 509-517.
26	Engage in dialogue	Dialogue	21	Dunston, R., Lee, A., Boud, D., Brodie, P., & Chiarella, M. (2009). Co-production and health system reform–from re-imagining to re-making. Australian Journal of Public Administration, 68(1), 39-52.
27	Learning	Learning	23	Egid, B. R., Roura, M., Aktar, B., Quach, J. A., Chumo, I., Dias, S., ... & Ozano, K. (2021). ‘You want to deal with power while riding on power’: global perspectives on power in participatory health research and co-production approaches. BMJ global health, 6(11), e006978.
28	Reasoning	Reasoning	1	Alford, J. (2009). Engaging public sector clients: From service-delivery to co-production. Springer.

28	Reasoning	Reasoning	19	Donetto, S., Pierri, P., Tsianakas, V., & Robert, G. (2015). Experience-based co-design and healthcare improvement: realizing participatory design in the public sector. The Design Journal, 18(2), 227-248.
28	Reasoning	Reasoning	31	Howarth, C., Lane, M., Morse-Jones, S., Brooks, K., & Viner, D. (2022). The 'co'in co-production of climate action: challenging boundaries within and between science, policy and practice. Global Environmental Change, 72, 102445.
28	Reasoning	Reasoning	39	Osborne, S. P., & Strokosch, K. (2013). It takes Two to Tango? Understanding the C o-production of Public Services by Integrating the Services Management and Public Administration Perspectives. British Journal of Management, 24, S31-S47.
28	Reasoning	Reasoning	47	Ramirez, R. (1999). Value co-production: intellectual origins and implications for practice and research. Strategic management journal, 20(1), 49-65.
29	Reciprocate	Reciprocity	14	Cahn, E. S. (2000). No more throw-away people: The co-production imperative. Edgar Cahn.
29	Reciprocate	Reciprocity	23	Egid, B. R., Roura, M., Aktar, B., Quach, J. A., Chumo, I., Dias, S., ... & Ozano, K. (2021). 'You want to deal with power while riding on power': global perspectives on power in participatory health research and co-production approaches. BMJ global health, 6(11), e006978.
29	Reciprocate	Reciprocity	36	Needham, C., & Carr, S. (2009). SCIE research briefing 31: co-production: an emerging evidence base for adult social care transformation. London: Social Care Institute for Excellence.
29	Reciprocate	Reciprocity	52	SCIE (2013, 2022) Co-production: what it is and how to do it. https://www.scie.org.uk/co-production/what-how (accessed November 21st 2024).
29	Reciprocate	Reciprocity	52	SCIE (2013, 2022) Co-production: what it is and how to do it. https://www.scie.org.uk/co-production/what-how (accessed November 21st 2024).
29	Reciprocate	Reciprocity	53	Staniszewska, S., Hickey, G., Coutts, P., Thurman, B., & Coldham, T. (2022). Co-production: a kind revolution. Research Involvement and Engagement, 8(1), 4.
29	Reciprocate	Reciprocity	54	Slay, J., & Stephens, L. (2013). Co-production in mental health: A literature review. London: new economics foundation, 4, 1-36.
30	Reflect	Reflexivity	25	Gibbons, M., Limoges, C., Scott, P., Schwartzman, S., & Nowotny, H. (1994). The new production of knowledge: The dynamics of science and research in contemporary societies.
30	Reflect	Reflexivity	26	Gillard, S., Simons, L., Turner, K., Lucock, M., & Edwards, C. (2012). Patient and public involvement in the coproduction of knowledge: reflection on the analysis of qualitative data in a mental health study. Qualitative Health Research, 22(8), 1126-1137.
30	Reflect	Reflexivity	28	Green, R., & Baker, C. (2022). Re-empowering into voice: experiments in organic community co-production. Community Development Journal, 57(2), 277-294.
30	Reflect	Reflexivity	50	Rowley, H., Ivinson, G., Duggan, J., & Pahl, K. (2022). Editorial for SI–Critically exploring co-production. Qualitative Research Journal, 22(1), 1-6.

31	Respect	Respect	52	SCIE (2013, 2022) Co-production: what it is and how to do it. https://www.scie.org.uk/co-production/what-how (accessed November 21st 2024).
32	Share goals	Shared goals	3	Batalden, M., Batalden, P., Margolis, P., Seid, M., Armstrong, G., Opipari-Arrigan, L., & Hartung, H. (2016). Coproduction of healthcare service. BMJ quality & safety, 25(7), 509-517.
32	Share goals	Shared goals	15	Clark, M. (2015). Co-production in mental health care. Mental Health Review Journal, 20(4), 213-219.
33	Share knowledge	Sharing knowledge	4	Bate, P., & Robert, G. (2006). Experience-based design: from redesigning the system around the patient to co-designing services with the patient. BMJ quality & safety, 15(5), 307-310.
33	Share knowledge	Sharing knowledge	5	Bate, P., & Robert, G. (2007). Bringing User Experience to Healthcare Improvement: The Concepts, Methods and Practices of Experience-Based Design. Radcliffe.
33	Share knowledge	Sharing knowledge	8	Boyd H, McKernon S, Mullin B, Old A. (2012). Improving healthcare through the use of co-design. *NZ Med J*;125(1357):76-87.
33	Share knowledge	Sharing knowledge	15	Clark, M. (2015). Co-production in mental health care. Mental Health Review Journal, 20(4), 213-219.
33	Share knowledge	Sharing knowledge	17	Cooper, F., & Jones, C. (2022). Co-production for or against the university: student loneliness and the commodification of impact in COVID-19. Qualitative research journal, 22(1), 81-95.
33	Share knowledge	Sharing knowledge	34	The Point of Care Foundation. EBCD: Experience-based co-design toolkit. The Point of Care Foundation. Accessed July 14, 2020. https://www.pointofcarefoundation.org.uk/resource/experience-based-codesign-ebcd-toolkit
33	Share knowledge	Sharing knowledge	38	Nowotny, H., Scott, P. and Gibbons, M. (2001), Rethinking Science, Polity Press, Cambridge.
33	Share knowledge	Sharing knowledge	48	Realpe, A., & Wallace, L. (2010). What is Co-production? The Health Foundation.
33	Share knowledge	Sharing knowledge	49	Robert, G., Cornwell, J., Locock, L., Purushotham, A., Sturmey, G., & Gager, M. (2015). Patients and staff as codesigners of healthcare services. Bmj, 350.
33	Share knowledge	Sharing knowledge	51	Sanders, E. B. N., & Stappers, P. J. (2014). Probes, toolkits and prototypes: three approaches to making in codesigning. CoDesign, 10(1), 5-14.
33	Share knowledge	Sharing knowledge	54	Slay, J., & Stephens, L. (2013). Co-production in mental health: A literature review. London: new economics foundation, 4, 1-36.
34	Share leadership	Shared leadership	4	Bate, P., & Robert, G. (2006). Experience-based design: from redesigning the system around the patient to co-designing services with the patient. BMJ quality & safety, 15(5), 307-310.
35	Share ownership	Ownership	19	Donetto, S., Pierri, P., Tsianakas, V., & Robert, G. (2015). Experience-based co-design and healthcare improvement: realizing participatory design in the public sector. The Design Journal, 18(2), 227-248.
36	Share understanding	Shared understanding	23	Egid, B. R., Roura, M., Aktar, B., Quach, J. A., Chumo, I., Dias, S., ... & Ozano, K. (2021). ‘You want to deal with power while riding on power’: global perspectives on power in participatory health research and co-production approaches. BMJ global health, 6(11), e006978.
37	Take responsibility	Accountability	3	Batalden, M., Batalden, P., Margolis, P., Seid, M., Armstrong, G., Opipari-Arrigan, L., & Hartung, H. (2016). Coproduction of healthcare service. BMJ quality & safety, 25(7), 509-517.

37	Take responsibility	Accountability	15	Clark, M. (2015). Co-production in mental health care. Mental Health Review Journal, 20(4), 213-219.
37	Take responsibility	Accountability	18	Co-Production Collective (2022) Co-creating change together: Our direction for 2020 - 2022. London: Co-Production Collective, UCL
37	Take responsibility	Accountability	41	Osborne, S. (2010).The New Public Governance. New York, NY: Routledge.
37	Take responsibility	Accountability	53	Sharp, E. B. (1980). Toward a new understanding of urban services and citizen participation: The coproduction concept. Midwest Review of Public Administration, 14(2), 105-118.
38	Trust	Trust	7	Bovaird, T. (2007). Beyond engagement and participation: User and community coproduction of public services. Public administration review, 67(5), 846-860.
38	Trust	Trust	14	Cahn, E. S. (2000). No more throw-away people: The co-production imperative. Edgar Cahn.
38	Trust	Trust	37	Needham, C. (2008). Realising the potential of co-production: Negotiating improvements in public services. Social policy and society, 7(2), 221-231.
38	Trust	Trust	41	Osborne, S. (2010).The New Public Governance. New York, NY: Routledge.
39	Value all contributions	Value contribution	15	Clark, M. (2015). Co-production in mental health care. Mental Health Review Journal, 20(4), 213-219.
39	Value all contributions	Value contribution	28	Green, R., & Baker, C. (2022). Re-empowering into voice: experiments in organic community co-production. Community Development Journal, 57(2), 277-294.
39	Value all contributions	Value contribution	29	Liabo, K., Boddy, K., Bortoli, S., Irvine, J., Boult, H., Fredlund, M., ... & Morris, C. (2020). Public involvement in health research: what does ‘good’look like in practice?. Research involvement and engagement, 6, 1-12.
39	Value all contributions	Value contribution	48	Realpe, A., & Wallace, L. (2010). What is Co-production? The Health Foundation.
39	Value all contributions	Value contribution	52	SCIE (2013, 2022) Co-production: what it is and how to do it. https://www.scie.org.uk/co-production/what-how (accessed November 21st 2024).
40	Work together	Transdisciplinary	25	Gibbons, M., Limoges, C., Scott, P., Schwartzman, S., & Nowotny, H. (1994). The new production of knowledge: The dynamics of science and research in contemporary societies.
40	Work together	Transdisciplinary	26	Gillard, S., Simons, L., Turner, K., Lucock, M., & Edwards, C. (2012). Patient and public involvement in the coproduction of knowledge: reflection on the analysis of qualitative data in a mental health study. Qualitative Health Research, 22(8), 1126-1137.
